# Editorial: Advances in radiotherapy for prostate cancer

**DOI:** 10.3389/fonc.2022.1122652

**Published:** 2022-12-22

**Authors:** Sophia C. Kamran, Linda G.W. Kerkmeijer, Constantinos Zamboglou

**Affiliations:** ^1^Department of Radiation Oncology, Massachusetts General Hospital, Harvard Medical School, MA, Boston, United States; ^2^Broad Institute of MIT and Harvard, Cambridge, MA, Boston, United States; ^3^Department of Radiation Oncology, Radboud University Medical Center, Nijmegen, Netherlands; ^4^Department of Radiation Oncology, Faculty of Medicine, Medical Center - University of Freiburg, University of Freiburg, Freiburg, Germany; ^5^German Oncology Center, European University Cyprus, Limassol, Cyprus

**Keywords:** prostate cancer, radiation oncology, PSMA PET, stereotactic ablation body radiation therapy, oligometastasic prostate cancer, HDR brachytherapy, ARAT

## Introduction

Prostate cancer (PCa) is the second most frequent cancer diagnosis in men worldwide and radiation therapy (RT) is a main treatment option for all disease stages. Recent developments in diagnostic medical imaging and high-precision RT techniques ensure that PCa patients can be cured while maintaining an excellent quality of life. In parallel, a deeper understanding of tumor biology facilitates the evolution from a “one size fits all approach” to a personalized therapy.

The goal of this Research Topic was to concentrate excellent and multidisciplinary scientific contributions on the evolving field of RT for PCa patients. The Research Topic accepted 13 articles including a total of 126 authors, demonstrating the rapid scientific progress in this field. The manuscripts of the Research Topic can be divided into the following topics according to the patient’s disease stage.

## Topic 1: Primary localized PCa

Definitive RT for patients with primary localized PCa is a main treatment option providing an excellent tumor control and maintaining the patient’s quality of life. Current research for definitive RT for primary PCa patients includes the implementation of advanced imaging techniques for improved staging as well as focal dose escalation, the admission of new hormonal agents and the reduction of treatment time by delivering hypofractionated treatment regimen.

Two studies in the Research Topic evaluated the role of prostate specific membrane antigen positron emission tomography (PSMA-PET) for primary prostate cancer patients. Zschaeck et al. examined retrospectively in 135 patients with PCa, which quantitative PSMA-PET parameters have the highest correlation with clinical and histological tumor aggressiveness. The authors concluded that SUVmax values in PSMA-PET show a superiority for the detection of high-risk patients with AUC values up to 0.73. Marinescu et al. performed an intraindividual comparison between [18F] PSMA-1007 PET/CT and multiparametric magnetic resonance imaging (mpMRI) in 93 primary PCa patients and reported a significant influence of PSMA-PET imaging on radiotherapy target volumes and the patients’ cT stage; PSMA-PET detected significant more lesions and the PET-derived target volumes were significantly larger (2.05 *vs*. 3.65 ml, *p*<0.01). The authors concluded that combined mpMRI and PSMA-PET information should be used to guide focal dose escalation concepts.

Lara et al. presented the preliminary results of a prospective phase II study which assessed the effectiveness of 6 months enzalutamide monotherapy combined with hypofractionated external-beam radiotherapy (EBRT) for treating intermediate risk prostate cancer in 62 patients. The treatment was in general well tolerated with no significant changes in patients’ quality of life. However, severe grade 3 acute systemic toxicity related to hypertension was observed in 19/56 (34%) patients. All the patients showed a prostate specific antigen (PSA) response 6 months after the end of Enzalutamide treatment. In a cost utility analysis by Farah et al. the authors compared robot-assisted Radical Prostatectomies (rRP) and robot-assisted stereotactic body radiotherapy in France. Stereotactic body radiotherapy seemed to be more cost-effective than rRP in terms of quality-adjusted life years (8.37 vs 6.85) despite the slightly higher initial cost due to the use of RT.

Finally, Tamihardja et al. evaluated the clinical outcome of two-weekly high-dose-rate brachytherapy boost after EBRT for 338 patients with localized prostate cancer. After a median follow-up of 101.8 months the authors observed an excellent toxicity profile with low GU/GI toxicity rates and effective long-term biochemical control (76% after ten years).

## Topic 2: Elective nodal irradiation in patients submitted to radical, adjuvant or salvage RT

The role of elective nodal irradiation for intermediate/high/very-high risk non-metastatic prostate cancer is still debated. Thus, Guerini et al. present a preliminary analysis of the PRO-EP study, a multicenter observational study on Elective Pelvic nodes Irradiation (doi.org/10.3389/fonc.2022.951220). Forty-three centers enrolled 1029 patients. The follow-up, at this time, is too short to draw conclusions regarding cancer control outcomes, however, toxicity was mild, and there were no statistically significant differences in quality-of-life outcomes.

## Topic 3: (Oligo)metastasized prostate cancer

There has been a paradigm shift in the understanding of metastatic PCa, particularly in the setting of advanced molecular imaging. Historically, metastatic prostate cancer was treated with systemic therapy, however, in recent years, the emergence of the “oligometastatic” disease state has led to novel indications for RT. At this time, oligometastatic prostate cancer is defined clinically, and can be broadly bucketed into *de novo* oligometastatic disease, oligorecurrent disease, and oligoprogressive disease, as detailed by Yaney et al. in their mini-review. They also highlight the emerging data behind the benefit of RT in these three indications. The authors discuss the need for large, randomized trials to further clarify which patients will benefit from metastasis-directed therapy, alluding to ongoing studies currently underway.

The definition of oligoprogressive disease can be vexing, with little data on the significance of oligoprogression in patients with castrate-resistant disease on novel antiandrogen therapy (e.g. androgen receptor-targeted therapy, ARTT, such as enzalutamide or abiraterone). Patel et al. evaluated 102 patients with metastatic castrate-resistant PCa (mCRPC) on ARTT at a single institution, finding that thirty (29%) of patients presented with oligoprogression and 21 patients (21% of total) had lesions suitable for SBRT. Most lesions were in the bone (46%) or lymph nodes (33%). Median progression-free survival to oligoprogression versus polyprogression was 16.8 versus 11 months. Time to further progression after oligoprogression was 13.6 months among those who received SBRT, versus 5.7 months in those treated with continuation of ARTT alone.

Xu et al. evaluated the efficacy and safety of SBRT (using CyberKnife) for PCa oligometastases in China. Between May 2012 and February 2021, 75 patients with 108 oligometastases were treated. With a median follow-up time of 23.2 months, the complete response, partial response, stable disease, and progressive disease rates were 63.0%, 10.2%, 21.3%, and 5.6% respectively. Among those with metastatic castration-resistant PCa, the 2-year local control rates were 93.8%, while for the 60 metastatic hormone-sensitive prostate cancer patients, the 2-year local control rates were 96.7%. In 27 patients not on androgen deprivation therapy, 2-year freedom from ADT was 44.0%. This study determined that SBRT is safe and effective.

Due to advances in imaging, the utility of biology-guided radiotherapy (BgRT) is an attractive and novel therapeutic modality to guide radiation therapy based on functional imaging. This was explored by Gaudreault et al. using PSMA-PET. The team described nodal and distant metastatic distribution of lesions to determine the proportion of metastatic lesions suitable for BgRT. Using a single-institution patient subset from the ProPSMA trial, the team contoured gross tumor volumes (GTV) on the CT component of PSMA PET/CT scans. Lesions were considered suitable for biology-guided radiotherapy if 1) normalized SUV was larger than an nSUV threshold and 2) adjacent non-tumor tissue was free of PSMA-PET uptake inside the outer shell expansion. A majority of lesions evaluated were determined to be suitable for BgRT using a 10-mm tracking zone. Some lesions did have adjuvant non-tumor uptake, due to proximity of the ureter or bladder, and thus may require exclusion from emission tracking during BgRT. However, this represents a very novel technique to deliver treatment based on biological features of disease and incorporating this into radiation delivery, thus representing a potential role for efficient therapy of metastatic disease.

In patients who present with *de novo* lymph node-positive PCa, external beam radiation therapy (EBRT) + ADT is recommended as the preferred treatment option. Yet, the incorporation of PSMA-PET impacts EBRT treatment fields. The study by Spohn et al. sought to understand characteristics associated with biochemical recurrence after definitive radiotherapy when using PSMA-PET in the staging of clinically node positive patients. Forty-eight patients staged by PSMA-PET were included. All patients received EBRT to the pelvis +/- boost to positive nodes. With a median follow-up of 24 months, it was found that more than 2 PET-positive pelvic lymph nodes are associated with unfavorable biochemical recurrence-free survival, and high SUVmax values are associated with unfavorable metastasis-free survival. The authors suggest that these may be relevant prognostic factors to identify patients with favorable outcomes.

One important aspect when using high doses to treat the prostate, particularly in the setting of metastatic disease, is to ensure accurate visualization and targeting of delivery. Li et al. describe a case using daily MR-guided adaptive radiation therapy to treat a 65-year-old gentleman with metastatic PCa to his prostate. The patient received 36 Gy over 6 weekly fractions. The target volume had a marked 49% reduction – which was accounted for in the online adaptive process. This case report demonstrated the promising value for using the MR-linac for adaptive RT.

Cheng et al. then go on to describe a study protocol combining tislelizumab and multisite RT for patients with mCRPC. All patients had at least 1 site suitable for RT and failed ADT, followed by one novel second-line endocrine therapy. Patients received tislelizumab monotherapy induction therapy for two cycles, followed by one cycle combined with RT, followed by tislelizumab maintenance. The goal of this therapy is to potentially demonstrate a promising strategy for synergistic enhancement of treatment efficacy.

## Conclusion

As demonstrated by the vast amount of unique research articles as part of this topic, PCa research is rapidly advancing in all disease stages. Definitive RT has been established in the treatment of primary PCa. The role of RT in oligometastatic disease will be elucidated and continue to evolve in this disease space. The coming years will see advances in imaging, techniques, combination therapies, and improved personalization of therapies.

## Author contributions

All authors listed have made a substantial, direct, and intellectual contribution to the work and approved it for publication.

